# Influence Analysis of Digital Financial Risk in China's Economically Developed Regions Under COVID-19: Based on the Skew-Normal Panel Data Model

**DOI:** 10.3389/fpubh.2022.822097

**Published:** 2022-02-21

**Authors:** Rendao Ye, Yichen Xie, Na An, Ya Lin

**Affiliations:** School of Economics, Hangzhou Dianzi University, Hangzhou, China

**Keywords:** COVID-19, digital financial risk, AHP, entropy weight, skew-normal panel data model, EM algorithm

## Abstract

The rapid spread of COVID-19 worldwide makes an uncertain impact on the development of digital finance in China. In this background, the measurement of digital financial risk and analysis of influence factor become the focus of the financial field. Therefore, this article builds the indicator system of digital financial risk and uses the Lagrange multiplier method to obtain the optimal comprehensive weight of AHP and entropy weight. Then, this article measures the digital financial risk indexes of China's major regions with high-level economic development from 2013 to 2020. Furthermore, the maximum likelihood estimates of the unknown parameters of skew-normal panel data model are obtained based on the EM algorithm, and the comparative study of the normal and skew-normal panel data models is conducted under AIC and BIC. Finally, based on the result of the model, the influence factors of digital financial risk of China's economically developed regions under COVID-19 are analyzed to provide data support for the prevention and governance of digital financial risk.

## Introduction

With the outbreak of COVID-19, although China has taken various prevention and control measures to contain its spread, the development of finance is still affected to varying degrees, and digital finance is especially influenced, which may cause systemic digital financial risk. In this background, it is of great academic value and practical significance to measure the digital financial risk statistically and construct the statistical model to explore the influence factors of the risk.

At present, many scholars have studied digital financial risk from the aspects of business, indicator, and measurement. For example, Fang et al. (1) used the accelerator and feedback mechanisms of traditional financial systemic risk to analyze fintech systemic risk from the aspects of business and technology. Wu (2) and Wei (3) analyzed the risk of digital inclusive finance from the perspective of combining digital technology and inclusive finance and explored the regulatory problem brought by digital inclusive finance. Further, Zhang and Zhang (4) and Ma and Li (5), respectively, constructed an internet financial risk evaluation index system from five different dimensions and used the analytic hierarchy process (AHP) to measure internet financial risk. Lv (6) established the risk index system of online loan platforms and applied R-type cluster analysis to reduce the dimension of the index system and then used the DEA model to rate the efficiency of the platform's risk control ability. Based on this, Ouyang and Mo (7), and Wei (8), respectively, used the Pareto-type extreme-value distribution and Monte Carlo model to measure the risk of internet finance and proved the excellent fitting property of the model.

The existed models in applied fields often assume that random variables are normally distributed. However, the actual data more often and frequently show the characteristics of asymmetrically skew-normal distribution. Therefore, some scholars have further conducted researches on the theory and application of the skew-normal model. For example, Ye and Wang (9) gave the density function, moment generation function, and independence condition of the linear mixed models with skew-normal random effects and constructed the exact tests of fixed effects and variance components. Jin et al. (10) proved that the penalized maximum likelihood estimator is strongly consistent when the putative order of mixture is not less than the true one. Ye et al. (11), Meng and Xiao (12), respectively, explored the influence factors of the efficiency of China's green economy and measurement of credibility premium based on the skew-normal panel data model and skew-normal random effect model. Arellano-Valle et al. (13), Huang and Dagne (14), and Wang (15) applied the skew-normal linear mixed model to the case studies of cholesterol data set, HIV data, and insurance loss data, respectively, and found that the data are better fitted in this model under the assumption of the skew-normal distribution. However, the existed studies have not analyzed the influence factors of digital financial risk under the skew-normal model, which has become a focus of finance under COVID-19 especially. Therefore, this article measures the digital financial risk index under COVID-19 and studies the influence factors of digital financial risk in China's economically developed regions based on the skew-normal panel data model.

The structure of this article is as follows. In the second section, the Lagrange multiplier method is used to obtain the optimal comprehensive weight of AHP and entropy weight, and the digital financial risk indexes of China's major regions with high-level economic development from 2013 to 2020 are measured. In the third section, the normal and skew-normal panel data models are constructed and compared under Akaike information criterion (AIC) and Bayesian information criterion (BIC), and the influence factors of digital financial risk of China's major regions with high-level economic development under COVID-19 are analyzed. Additionally, the fourth section gives the conclusion and suggestion.

## Measurement of Digital Financial Risk

### Construction of Indicator System

Digital financial risk includes both traditional financial risk and internet technology risk. Therefore, combined with the characteristics of digital financial business, this article uses the risk category enumeration method to identify and summarize the potential risk types of digital finance of China's major regions with high-level economic development. Additionally, the indicator system of digital financial risk is constructed, which includes 5 first-level indicators and 12 second-level indicators, as shown in Table 1.

**Table 1:** Indicator system of digital financial risk of China's economically developed regions.

**First-level indicator**	**Second-level indicator**
Operation risk I_1_	Number of websites tampered with I_11_
	Number of websites implanted with backdoor I_12_
Credit risk I_2_	Times interest earned (TIE) of cash flow of digital finance enterprises I_21_
	Number of troubled enterprises in the current year I_22_
	Cumulative number of troubled enterprises in the current year I_23_
Market risk I_3_	Annualized yield of digital finance enterprises I_31_
	Growth rate of online retail sales I_32_
	Price-earnings ratio of digital finance enterprises I_33_
Liquidity risk I_4_	Turnover ratio of account payable of digital finance enterprises I_41_
	Net assets year-on-year growth of digital finance enterprises I_42_
	Acid test ratio of digital finance enterprises I_43_
Policy risk I_5_	Number of policies and regulations on digital financial issued by government departments I_51_

*^1^Operation risk. The network security risk may arise for the improper operations of digital finance enterprises or customers, which increases the probability of network vulnerabilities and makes network viruses easier to penetrate. Therefore, this article measures the operation risk of digital finance by the number of websites that are tampered with and the number of websites that are implanted with backdoor.*

*^2^Credit risk. For the lack of complete credit system, digital finance enterprises and customers have the default risk because of the lack of interest repayment ability and other problems. Consequently, this article employs the TIE of cash flow of digital finance enterprises and the number of troubled enterprises to measure the credit risk of digital finance.*

*^3^Market risk. For the low entry threshold of the digital financial market and the uneven scale and professional level of the digital financial market, this article uses the annualized yield and price-earnings ratio of digital finance enterprises to measure the risk of the digital financial market. In addition, because of the strong positive correlation between the e-commerce market and digital financial market, the growth rate of online retail sales is also included in the index to measure market risk.*

*^4^Liquidity risk. As digital finance enterprises have not established a complete financial security system, it is easy to encounter capital turnover difficulties. So, this article takes the accounts payable turnover rate, net assets year-on-year growth, and acid test ratio of digital finance enterprises as the indicators to measure the liquidity risk of digital finance.*

*^5^Policy risk. The digital financial supervision policy is not perfect at present, which causes the absence of digital financial supervision easily. Therefore, this article selects the number of regulatory policies or regulations on digital finance to measure its policy risk. The more the number of policies and regulations, the more complete supervision framework of the digital finance industry, which means the lower the risk of digital finance*.

### Method of Weight Determination

#### AHP Method

Based on the indicator system of digital financial risk of China's economically developed regions, we construct the goal layer, criteria layer, and subcriteria layer. The goal layer is digital financial risk, the criteria layer is 5 first-level indicators, and the subcriteria layer is 12 second-level indicators. Then, the judgment matrix is constructed as below.


$$\begin{array}{l} A={\left(a_{ij}\right)}_{n\times n}=\left[\begin{array}{cccc} a_{11} & a_{12} & \cdots & a_{1n}\\ a_{21} & a_{22} & \cdots & a_{2n}\\ \vdots & \vdots & \ddots & \vdots \\ a_{n1} & a_{n2} & \cdots & a_{nn} \end{array}\right], \end{array}$$

where the scale value *a*_*ij*_ represents the importance of index *i* relative to index *j*, *a*_*ij*_ > 0, $a_{ij}=\frac{1}{a_{ji}}$, and *a*_*ii*_ = 1. The scaling of *a*_*ij*_ refers to Saaty scale method (16), which is shown in Table 2.

**Table 2:** Saaty scale method.

**Scale**	**Implication**
1	Indicator *i* is as important as indicator *j*
3	Indicator *i* is slightly more important than indicator *j*
5	Indicator *i* is more important than indicator *j*
7	Indicator *i* is significantly more important than indicator *j*
9	Indicator *i* is absolutely more important than the indicator *j*
2, 4, 6, 8	Intermediate scale values between the adjacent scales above
Reciprocal	If the scale value *a*_*ij*_ = *n*, then the scale value *a*_*ji*_ = 1/*n*

To calculate the indicator weight, we normalize each column in the judgment matrix *A* to get ${ã}_{ij}=a_{ij}/\overset{n}{\underset{j=1}{\sum }}a_{ij}$, sum the rows of ã_*ij*_ and get ${\tilde{p}}_{i}=\overset{n}{\underset{i=1}{\sum }}{ã}_{ij}$, and normalize ${\tilde{p}}_{i}$ to obtain the index subjective weight $p_{i}={\tilde{p}}_{i}/\overset{n}{\underset{i=1}{\sum }}{\tilde{p}}_{i}$ and the weight vector (*p*_1_, *p*_2_, ... , *p*_n_)′.

Then, to test the consistency of the judgment matrix, we obtain the maximal eigenroot λ_max_ of the judgment matrix *A* by *AP* = λ_max_*P*, calculate the consistency index *CI*=$\frac{\lambda_{\max}-n}{n-1}$, and get the consistency ratio by *CR* = *CI*/*RI*. Here, *RI* is the random consistency indicator (16), as shown in Table 3.

**Table 3:** Value of the random consistency indicator *RI*.

**n**	**1**	**2**	**3**	**4**	**5**	**6**	**7**	**8**	**9**	**10**
RI	0	0	0.58	0.9	1.12	1.24	1.32	1.41	1.45	1.49

Particularly, if the consistency ratio *CR* < 0.1, then the judgment matrix *A* meets the consistency condition. If *CR* > 0.1, then the judgment matrix *A* needs to be adjusted until it meets the condition.

#### Entropy Weight Method

The entropy weight method is an objective method that determines the weight of the indicator according to the variability. If the information entropy of the indicator is smaller, the difference degree of the indicator value is larger, the information provided is more, and thus, the weight is larger.

First, due to the different dimensions of indicators, it is necessary to standardize the original index data matrix *X* = (_*x*_*ij*_)*n* × *m*_ of *n* indicators and *m* years. If the *i*-th indicator is a positive indicator, then


$$\begin{array}{l} {x_{ij}}^{*}=\frac{x_{i\max}-x_{ij}}{x_{i\max}-x_{i\min}}. \end{array}$$

If the *i*-th indicator is a negative indicator, then


$$\begin{array}{l} x_{ij}^{*}=\frac{x_{ij}-x_{i\min}}{x_{i\max}-x_{i\min}}, \end{array}$$

where *x*_*i*min_ and *x*_*i*max_ are the minimum value and maximum value of the indicator *i* in the *m* years, respectively. Further, we calculate the proportion of the index *i* in the *j*-th year


$$\begin{array}{l} {\tilde{q}}_{ij}=\frac{x_{ij}^{*}}{\overset{m}{\underset{j=1}{\sum }}x_{ij}^{*}} \end{array}$$

and the entropy value of the index *i*


$$\begin{array}{l} e_{i}=-\frac{1}{\ln\;m}\overset{m}{\underset{j=1}{\sum }}{\tilde{q}}_{ij}\ln\;{\tilde{q}}_{ij}. \end{array}$$

Finally, we obtain the weight coefficient of the index *i*


$$\begin{array}{l} q_{i}=\frac{1-e_{i}}{n-\overset{n}{\underset{i=1}{\sum }}e_{i}}. \end{array}$$

#### Comprehensive Weight Method

The comprehensive weight method not only overcomes the subjective defect of AHP, but also makes up for the lack of professionalism of the entropy weight method. First, we calculate the comprehensive weight $p_{i}^{*}$ based on the subjective weight *p*_*i*_ obtained by AHP and objective weight *q*_*i*_ obtained by the entropy weight method


$$\begin{array}{l} p_{i}^{*}=\frac{p_{i}q_{i}}{\overset{n}{\underset{i=1}{\sum }}p_{i}q_{i}},i=1,2,\cdots \;,n. \end{array}$$

Then, to make $p_{i}^{*}$ as close to *p*_*i*_ and *q*_*i*_ as possible, we construct the objective function based on the principle of minimum information entropy


$$\begin{array}{l} \text{min }E=\sum_{i=1}^{n}p_{i}^{*}(\ln\frac{p_{i}^{*}}{p_{i}})+\sum_{i=1}^{n}p_{i}^{*}(\ln\frac{p_{i}^{*}}{q_{i}}),i=1,2,\cdots ,n. \end{array}$$

Finally, the optimized comprehensive weight is obtained by the Lagrange multiplier method


$$\begin{array}{l} p_{i}^{*}=\frac{{\left(p_{i}q_{i}\right)}^{\frac{1}{2}}}{\overset{n}{\underset{i=1}{\sum }}{\left(p_{i}q_{i}\right)}^{\frac{1}{2}}},i=1,2,\cdots \;,n, \end{array}$$

where $\overset{n}{\underset{i=1}{\sum }}p_{i}^{*}=1,\;p_{i}^{*}>0$.

### Measurement of the Digital Financial Risk Index

#### Data Selection

This article selects the annual data of China's major regions with high-level economic development, Beijing, Shanghai, Zhejiang, and Jiangsu^1^ from 2013 to 2020 for a total of 8 years. The operation risk data come from *China Internet Network Security Report*, and the data of credit risk, market risk, and liquidity risk come from Flush, Wangdaizhijia, and the statistical yearbooks of the above four regions, and the policy risk data come from government websites.

#### Indicator Weight Determination

First, the weight of each indicator under AHP is calculated. According to the principle of AHP, the weight of each indicator *p*_*i*_ is calculated, and the consistency test results show that the *CR* of all indicators are < 0.1, and thus, the weights are considered to have passed the test. The weights of indicators are shown in Table 4.

**Table 4:** Weights of indicators under AHP *p*_*i*_(*i* = 1, 2, ⋯ , 12).

**Criteria layer**	**Weight**	**Subcriteria layer**	**Weight**	***p*_*i*_**
I_1_	0.1046	I_11_	0.6667	0.0697
		I_12_	0.3333	0.0349
I_2_	0.4142	I_21_	0.7226	0.2993
		I_22_	0.1741	0.0721
		I_23_	0.1033	0.0428
I_3_	0.1841	I_31_	0.2973	0.0547
		I_32_	0.1638	0.0302
		I_33_	0.5389	0.0992
I_4_	0.2625	I_41_	0.2973	0.0780
		I_42_	0.1638	0.0430
		I_43_	0.5389	0.1415
I_5_	0.0346	I_51_	0.0346	0.0346

Second, the weight of each indicator under the entropy weight method is calculated. Finally, the subjective weight under the AHP is modified to obtain the comprehensive indicator weight, as shown in Table 5.

**Table 5:** Weights under entropy weight method *q*_*i*_ and comprehensive weights $p_{i}^{*}\left(i\;=\;1,2,\cdots \;,12\right)$.

**Second-level indicator weight**	**Beijing**		**Shanghai**		**Zhejiang**		**Jiangsu**	
	***q*_*i*_**	${\text{p}}_{i}^{*}$	***q*_*i*_**	${\text{p}}_{i}^{*}$	***q*_*i*_**	${\text{p}}_{i}^{*}$	***q*_*i*_**	${\text{p}}_{i}^{*}$
I_11_	0.1884	0.1391	0.1350	0.1054	0.1888	0.1292	0.1381	0.1058
I_12_	0.0868	0.0668	0.0765	0.0561	0.0862	0.0617	0.0826	0.0579
I_21_	0.0371	0.1278	0.0888	0.1771	0.0352	0.1155	0.0616	0.1464
I_22_	0.0996	0.1029	0.0991	0.0918	0.1251	0.1069	0.0847	0.0843
I_23_	0.0967	0.0781	0.0762	0.0620	0.0908	0.0702	0.0775	0.0621
I_31_	0.0559	0.0671	0.0801	0.0719	0.0645	0.0669	0.0469	0.0546
I_32_	0.0459	0.0452	0.0370	0.0363	0.0965	0.0607	0.0886	0.0557
I_33_	0.0624	0.0955	0.2381	0.1669	0.1203	0.1230	0.1069	0.1110
I_41_	0.1166	0.1158	0.0724	0.0817	0.0519	0.0716	0.1276	0.1076
I_42_	0.0615	0.0624	0.0304	0.0393	0.0379	0.0455	0.0499	0.0499
I_43_	0.0474	0.0994	0.0341	0.0755	0.0658	0.1086	0.0908	0.1222
I_51_	0.1018	0.0720	0.0321	0.0362	0.0369	0.0402	0.0448	0.0425

#### Index Measurement

To calculate the digital financial risk indexes of the four regions of Beijing, Shanghai, Zhejiang, and Jiangsu *Y*_1_, *Y*_2_, *Y*_3_ and *Y*_4_ from 2013 to 2020, the comprehensive weight in Table 5 is multiplied by the value $X_{a}^{*}$ after the standardized treatment, then we obtain that


$$\begin{array}{l} Y_{a}=P_{a}^{*}X_{a}^{*},a=1,2,3,4, \end{array}$$

where $P_{a}^{*}=\left[p_{11}^{*},p_{12}^{*},\ldots ,p_{112}^{*}\right]$, $X_{a}^{*}={\left(x_{ij}^{*}\right)}_{12\times 8}=\left[\begin{array}{cccc} x_{11}^{*} & x_{12}^{*} & \cdots & x_{18}^{*}\\ x_{21}^{*} & x_{22}^{*} & \cdots & x_{28}^{*}\\ \vdots & \vdots & \ddots & \vdots \\ x_{121}^{*} & x_{122}^{*} & \cdots & x_{128}^{*} \end{array}\right]$,*Y*_*a*_=[*Y*_11_, *Y*_12_, …, *Y*_18_]. The calculation results are shown in Table 6.

**Table 6:** Digital financial risk indexes of China's major regions with high-level economic development.

	**2013**	**2014**	**2015**	**2016**	**2017**	**2018**	**2019**	**2020**
Beijing	0.3561	0.3529	0.3382	0.6562	0.5523	0.7485	0.9286	0.6492
Shanghai	0.4261	0.3356	0.3810	0.5209	0.3374	0.4504	0.5440	0.4476
Zhejiang	0.4279	0.3444	0.4283	0.4362	0.5658	0.6283	0.6249	0.5948
Jiangsu	0.4119	0.3687	0.4594	0.5451	0.5943	0.5860	0.7714	0.6888

**Figure 1:**
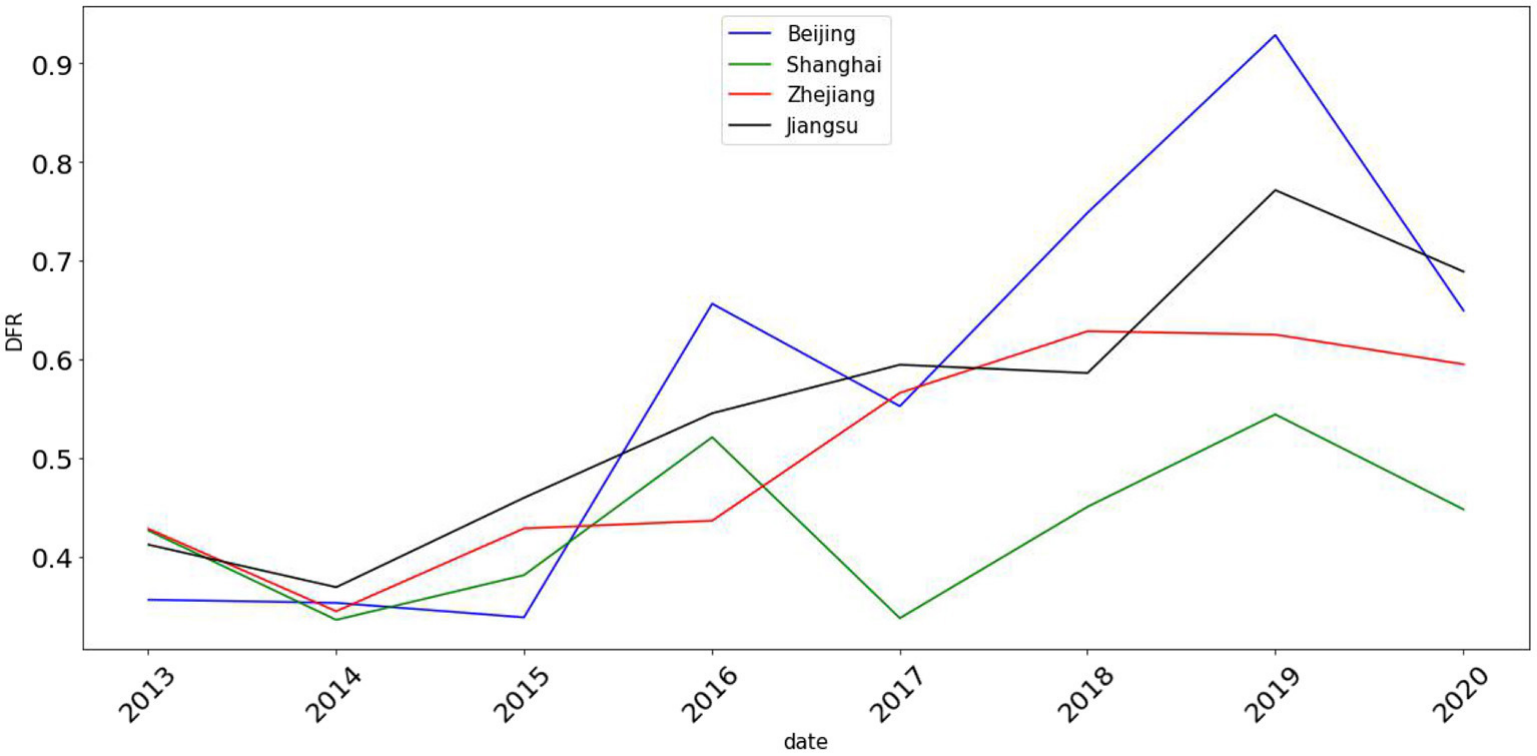
Line chart of digital financial risk indexes of China's major regions with high-level economic development from 2013 to 2020.

To further investigate the impact of COVID-19 on digital financial risk, this article takes December 31, 2019 as the starting point of COVID-19 and presents the line chart of digital financial risk indexes of four regions from 2013 to 2020, as pictured in Figure 1.

From Figure 1, before the outbreak of COVID-19, digital financial risk in all regions showed an overall upward trend. In 2016, due to the influence of policies and regulations of the digital finance industry, the values of digital financial risk in some regions showed a downward trend. After the outbreak of COVID-19, to continuously improve its digital operation and management capabilities and promote the sound development of the digital finance industry, the industry has made full use of its prominent advantages in online channels, full-time services, operation platforms, and process automation. Therefore, digital financial risk in all regions has gradually decreased.

## Influence Analysis of Digital Financial Risk Under Covid-19

### Test of Skew-Normal Distribution

Let *M*_*n*×*n*_ represent the set of all matrices *n*×*n* in the real number field *R*, and $R^{n}=M_{n\times 1}$. From Azzalini and Valle (17) and Azzalini and Capitanio (18), if the *n*-dimensional random vector Y follows the skew-normal distribution with location parameter μ, scale parameter Σ and skewness parameter α, which can be denoted as *Y*~*SN*_*n*_(μ, Σ, α), where μ∈*R*^*n*^, Σ∈*M*_*n*×*n*_, α∈*R*^*n*^. Then, the density function is


$$\begin{array}{l} f\left(x;\mu ,\Sigma ,\lambda \right)=2\varphi_{n}\left(x;\mu ,\Sigma \right)\Phi \left(\alpha^{T}\Sigma^{-1/2}\left(x-\mu \right)\right),x\in R^{n}, \end{array}$$

where φ_*n*_(*x*; μ, Σ) represents the *n*-dimensional normal density function with mean value μ and covariance matrix Σ, and Φ(·) represents the cumulative distribution function of the standard normal distribution. When α = 0, the skew-normal distribution degenerates into the normal distribution.

**Figure 2:**
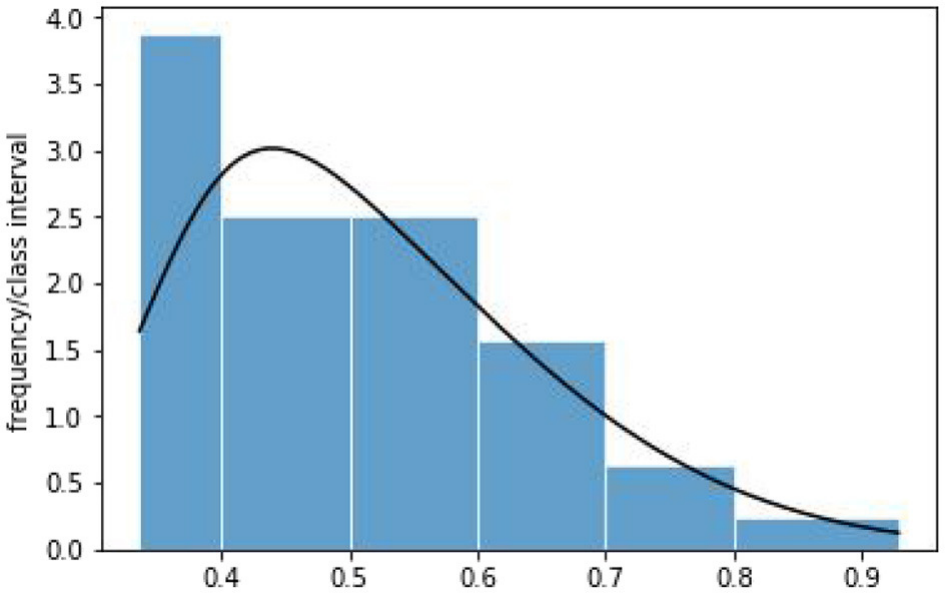
Histogram and probability density curve of digital financial risk indexes of China's major regions with high-level economic development.

This article first verifies the skew-normal distribution of the data including 32 digital financial risk indexes of Beijing, Shanghai, Zhejiang, and Jiangsu. The histogram of digital financial risk indexes is given in Figure 2. For testing the normality of the data, the *p*-values for Shapiro–Wilk, Kolmogorov–Smirnov, and Shapiro–Franci test are 0.0363, 6.8635e-13, and 0.0456, respectively. We can conclude that the data are not normally distributed at 5% significance level. Also, the chi-square goodness-of-fit test is used to test the null hypothesis that the data are skew-normally distributed. The value of the test statistic is χ^2^ = 0.4661, which is less than $\chi_{2}^{2}\;\left(0.05\right)=5.9915$, so the null hypothesis is not rejected at 5% significance level. Hence, the distribution of the digital financial risk indexes can be considered approximately skew-normal. Based on the method of moment estimation, the data are approximately distributed as SN (0.3405, 0.2275^2^, 3.6801) and its density curve is given in Figure 2.

#### Construction of Skew-Normal Panel Data Model Setting

To study the influencing factors of digital financial risk under COVID-19, this article selects the impact of COVID-19, digital finance development level, population size, fixed asset investment level, national economic development level and inflation level as independent variables, and regional digital financial risk index as dependent variable to construct the model, as shown in Table 7.

**Table 7:** Variable description.

**Type**	**Variable**	**Description**	**Abbreviation**
Dependent variable	Regional digital financial risk level	Digital financial risk index	*DFR*
Independent variable	Impact of COVID-19	Whether after the outbreak of COVID-19	*COVID-19*
	Digital finance development level	Logarithm of the digital financial inclusion index	ln *DFD*
	Population size	Logarithm of the population	ln *POP*
	Fixed asset investment level	Growth rate of fixed asset investment	*FAI*
	National economic development level	Growth rate of GDP	*GDP*
	Inflation level	Consumer price index-100	*INF*

*Data are from the Peking University digital financial inclusion index (2011–2020) report, national bureau of statistics, local bureau of statistics, and provincial national economic reports from 2013 to 2020*.

The development level of digital finance reflects the comprehensive development degree of digital inclusive finance in the region, and the size of population indicates the development potential of digital finance in the region. Because these two variables are large in value, logarithms are taken to reduce the heteroscedasticity of the model. The level of fixed asset investment reflects the development of regional investment. Reasonable fixed asset investment can effectively promote the regional economic growth and healthy development of digital financial platforms. The levels of national economic development and inflation reflect the investment enthusiasm of local residents and further indicate the investment level of the digital financial platform.

For the skew-normal characteristic of digital financial index, this article constructs the skew-normal panel data model to investigate the influencing factors of digital financial index of China's major regions with high-level economic development. The specific model is as follows


(1) $$\begin{array}{r} y_{it}=\beta_{0}+COVID-19_{it}\beta_{1}+\ln\;DFD_{it}\beta_{2}+\ln\;POP_{it}\beta_{3}\\ +FAI_{it}\beta_{4}+GDP_{it}\beta_{5}+INF_{it}\beta_{6}+\mu_{i}+\varepsilon_{it} \end{array}$$

where *y*_*it*_ represents the digital financial risk index of region *i* (*i* = 1, 2, 3, 4) and year *t* (*t* = 1, 2, ⋯ , 8), and COVID-19, ln *DFD*, ln *POP*, *FAI*, *GDP*, and *INF*, respectively, represent the impact of COVID-19, digital financial development level, population size, fixed asset investment level, national economic development level, and inflation level. β_0_ is the intercept, β_*j*_ (*j* = 1, 2, ⋯ , 6) is the regression coefficient, μ_*i*_ is the individual effect, and ε_*it*_ is the random error.


$$\begin{array}{r} Let\;Y=(y_{11},\ldots ,y_{18},y_{21},\ldots ,y_{28},\ldots ,y_{48}{)}^{\prime },\\ X=({X^{\prime }}_{1},{X^{\prime }}_{2},{X^{\prime }}_{3},{X^{\prime }}_{4}{)}^{\prime },\\ X_{i}=\left[\begin{array}{cccc} COVID\text{​}-\text{​}19_{11} & \ln DFD_{11} & \cdots & INF_{11}\\ COVID\text{​}-\text{​}19_{12} & \ln DFD_{12} & \cdots & INF_{12}\\ \vdots & \vdots & \ddots & \vdots \\ COVID\text{​}-\text{​}19_{18} & \ln DFD_{18} & \cdots & INF_{18} \end{array}\right],\\ \beta =(\beta_{1},\beta_{2},\ldots ,\beta_{6}{)}^{\prime },\mu ={\left(\mu_{1},\mu_{2},\mu_{3},\mu_{4}\right)}^{\prime }, \end{array}$$

and $\varepsilon ={\left(\varepsilon_{11},\ldots ,\varepsilon_{18},\varepsilon_{21},\ldots \varepsilon_{28},\ldots ,\varepsilon_{48}\right)}^{\prime }$, then Equation (1) can be written in matrix form as follows


$$\begin{array}{l} Y=1_{32}\beta_{0}+X\beta +\left(I_{4}⊗1_{8}\right)\mu +\varepsilon , \end{array}$$

where $\mu {\sim N}_{4}\left(0,\sigma_{1}^{2}I_{4}\right)$, $\varepsilon \sim SN_{32}\left(0,\sigma_{0}^{2}I_{32},\alpha \right)$, μ and ε are independent of each other.

#### Parameter Estimation

In this article, the digital financial risk index of China's major regions with high-level economic development is modeled and analyzed under the condition that the random error follows the normal distribution and skew-normal distribution, respectively. In addition, the maximum likelihood estimates of unknown parameters in the skew-normal panel data model are given based on the expectation-maximization (EM) algorithm (11), and then, the corresponding logarithmic likelihood values, AIC, and BIC are obtained. The results of parameter estimation are shown in Table 8.

**Table 8:** Parameter estimation results of normal and skew-normal panel data models.

**Variable**	**Normal panel data model**	**Skew-normal panel data model**
Intercept (β_0_)	−1.7617	−1.9378
*COVID-19* (β_1_)	−0.1046	−0.0970
*ln DFD* (β_2_)	0.2669	0.3047
*ln POP* (β_3_)	0.1046	0.0939
*FAI* (β_4_)	−0.0114	−0.0096
*GDP* (β_5_)	−0.0128	−0.0100
*INF* (β_6_)	0.0350	0.0424
Log-likelihood	34.2	58.1
AIC	−54.4	−102.2
BIC	−30.5	−78.3

From Table 8, under the condition that the random error follows the skew-normal distribution, the logarithmic likelihood value of the model is higher than that of the normal model, and AIC and BIC values are less than those of the normal model. Based on the above results, we know that the skew-normal panel data model is superior to the normal one under the AIC and BIC.

According to the simulated skew-normal panel data model, the coefficients of ln *DFD*, ln *POP*, *INF*, COVID-19, *FAI*, and *GDP* are 0.3047, 0.0939, 0.0424, −0.0970, −0.0096, and −0.0100, respectively. The results show that the development levels of digital finance and population size have positive impacts on the digital financial risk index. The existing digital finance industry has a low threshold and an imperfect regulatory system. Therefore, the potential risk of digital finance is continuously accumulated with the rapid development of digital finance and continuous expansion of population size. Moreover, the inflation level positively influences the digital financial risk index, which shows that the rise of regional inflation will raise the digital financial risk because of the increase of cost and operating pressure of some digital finance enterprises.

In contrast, the level of fixed asset investment and level of national economic development have negative impacts on the digital financial risk index. It shows that reasonable fixed asset investment and good national economic development level can reduce the liquidity risk of the digital finance industry. In addition, the impact of COVID-19 negatively influences the digital financial risk index. The reasons can be concluded as follows. First, financial customers have gradually increased the use of online financial products during the COVID-19, improving their operation level. As a result, the operation risk of the digital finance industry has been reduced. Second, the more demand for intelligent production and online office promotes the development of digital finance enterprises, increasing the liquidity funds. So, the liquidity risk of the digital finance industry is reduced. Finally, the outbreak of COVID-19 has made the government pay more attention to macroregulation of the digital finance industry by formulating some vigorous regulations and policies. Therefore, the policy risk of the digital finance industry is reduced. For example, successive regulations have made clear provisions on loans and deposits of the digital finance industry since 2020, which include the Interim Measures for the Administration of Commercial Bank Internet Loans, Interim Measures for the Administration of Online Micro-finance Business, and Deposits on Third-Party Internet Platforms.

## Conclusion and Suggestion

This article investigates the influencing factors of digital financial risks of China's economically developed regions under COVID-19, which aims to provide data support for China's digital financial risk prevention and governance practices. First, the financial risk index system of China's economically developed regions is constructed from the five aspects of operation risk, credit risk, market risk, liquidity risk, and policy risk. The Lagrange multiplier method is used to obtain the optimal comprehensive weight of AHP and entropy weight method, and then, we measure the digital financial risk indexes of China's economically developed regions from 2013 to 2020. The results show that the level of digital financial risk index presents a downward trend under COVID-19. Second, the maximum likelihood estimates of unknown parameters of the skew-normal panel data model are obtained based on the EM algorithm, and the normal and skew-normal panel data models are compared under the AIC and BIC, which indicate that the skew-panel data model fits better. Finally, the results show that the level of digital finance development, population size, and inflation level have positive impacts on the digital financial risk, whereas the impacts of COVID-19, fixed asset investment level, and national economic development level are negative.

Based on the above analysis, this article gives the following suggestions. First, Chinese government should improve the digital financial regulatory mechanism and credit system. Strengthening the standard construction of the digital finance industry can reduce the policy risk and credit risk of digital finance. Second, it is suggested to take the concept of financial digitization one step further. Taking COVID-19 as an opportunity, the digital finance industry should popularize the concept of digital finance to customers actively to promote its development and reduce the operational risks for customers. Third, it is essential for the financial industry to accelerate the digital transformation. Upgrading internet technology and financial application software development capabilities can accelerate the application of advanced technology, improve the risk management mechanism of financial institutions, and respond to unexpected public health events actively.

## Data Availability Statement

The original contributions presented in the study are included in the article/supplementary material, further inquiries can be directed to the corresponding author/s.

## Author Contributions

All authors listed have made a substantial, direct, and intellectual contribution to the work and approved it for publication.

## Funding

This research was supported by National Social Science Foundation of China under grant number 21BTJ068.

## Conflict of Interest

The authors declare that the research was conducted in the absence of any commercial or financial relationships that could be construed as a potential conflict of interest.

## Publisher's Note

All claims expressed in this article are solely those of the authors and do not necessarily represent those of their affiliated organizations, or those of the publisher, the editors and the reviewers. Any product that may be evaluated in this article, or claim that may be made by its manufacturer, is not guaranteed or endorsed by the publisher.

## Footnotes

^1^Beijing, Shanghai, Jiangsu, Fujian, Tianjin, and Zhejiang topped the list of the top six provinces in terms of per capita GDP in 2020 released by the National Bureau of Statistics. For the data missing of Tianjin and Fujian, this article only conducts the research on the four provinces of Beijing, Shanghai, Jiangsu, and Zhejiang.
